# Nanoporous Hollow Carbon Spheres Derived from Fullerene Assembly as Electrode Materials for High-Performance Supercapacitors

**DOI:** 10.3390/nano13050946

**Published:** 2023-03-05

**Authors:** Lok Kumar Shrestha, Zexuan Wei, Gokulnath Subramaniam, Rekha Goswami Shrestha, Ravi Singh, Marappan Sathish, Renzhi Ma, Jonathan P. Hill, Junji Nakamura, Katsuhiko Ariga

**Affiliations:** 1 International Center for Materials Nanoarchitectonics (WPI-MANA), National Institute for Materials Science (NIMS), 1-1 Namiki, Tsukuba 305-0044, Ibaraki, Japan; 2 Department of Materials Science, Faculty of Pure and Applied Sciences, University of Tsukuba 1-1-1, Tennodai, Tsukuba 305-8573, Ibaraki, Japan; 3 Department of Advanced Materials Science, Graduate School of Frontier Sciences, The University of Tokyo, 5-1-5 Kashiwanoha, Kashiwa 277-8561, Chiba, Japan; 4 Electrochemical Power Sources Division, CSIR-Central Electrochemical Research Institute, Karaikudi 630003, Tamil Nadu, India; 5 Academy of Scientific and Innovative Research (AcSIR), Ghaziabad 201002, Tamil Nadu, India; 6 Mitsui Chemicals, Inc., Carbon Neutral Research Center (MCI–CNRC), International Institute for Carbon-Neutral Energy Research (I2CNER), Kyushu University, 744 Motooka, Nishi-ku, Fukuoka-shi 819-0395, Fukuoka, Japan

**Keywords:** fullerene, self-assembly, hollow spheres, nanoporous carbons, supercapacitor

## Abstract

The energy storage performances of supercapacitors are expected to be enhanced by the use of nanostructured hierarchically micro/mesoporous hollow carbon materials based on their ultra-high specific surface areas and rapid diffusion of electrolyte ions through the interconnected channels of their mesoporous structures. In this work, we report the electrochemical supercapacitance properties of hollow carbon spheres prepared by high-temperature carbonization of self-assembled fullerene-ethylenediamine hollow spheres (FE-HS). FE-HS, having an average external diameter of 290 nm, an internal diameter of 65 nm, and a wall thickness of 225 nm, were prepared by using the dynamic liquid-liquid interfacial precipitation (DLLIP) method at ambient conditions of temperature and pressure. High temperature carbonization (at 700, 900, and 1100 °C) of the FE-HS yielded nanoporous (micro/mesoporous) hollow carbon spheres with large surface areas (612 to 1616 m^2^ g^−1^) and large pore volumes (0.925 to 1.346 cm^3^ g^−1^) dependent on the temperature applied. The sample obtained by carbonization of FE-HS at 900 °C (FE-HS_900) displayed optimum surface area and exhibited remarkable electrochemical electrical double-layer capacitance properties in aq. 1 M sulfuric acid due to its well-developed porosity, interconnected pore structure, and large surface area. For a three-electrode cell setup, a specific capacitance of 293 F g^−1^ at a 1 A g^−1^ current density, which is approximately 4 times greater than the specific capacitance of the starting material, FE-HS. The symmetric supercapacitor cell was assembled using FE-HS_900 and attained 164 F g^−1^ at 1 A g^−1^ with sustained 50% capacitance at 10 A g^−1^ accompanied by 96% cycle life and 98% coulombic efficiency after 10,000 consecutive charge/discharge cycles. The results demonstrate the excellent potential of these fullerene assemblies in the fabrication of nanoporous carbon materials with the extensive surface areas required for high-performance energy storage supercapacitor applications.

## 1. Introduction

Supercapacitors are currently promising electrochemical energy storage systems with advantages including extremely high-power densities, ultra-rapid charging rates, outstanding cycle lives and rate performances, easy operation, and low fabrication costs [1,2,3,4,5,6,7]. As a result, supercapacitors have been applied in electronic devices, hybrid vehicles, and for instant power backup applications. Porous carbon materials obtained from fullerenes by high temperature carbonization are expected to exhibit superior electrochemical energy storage performance over conventional carbon materials because of their large surface areas, improved electrical conductivities, well-defined pore size distributions, and their hierarchal and interconnected pore architectures [8,9,10,11]. In contrast to the two-dimensional (2D) reduced graphene oxide, a well-known and extensively explored electrode material, the carbon materials derived from essentially zero-dimensional (0D) fullerenes do not suffer from the aggregation or restacking processes commonly associated with graphene-like sheets. The uniqueness of fullerenes originates from the properties of their self-assemblies since, in contrast to other molecular-level nanocarbons, including carbon nanotubes (1D) or graphene or graphene oxides (2D), 0D fullerenes function as unique building blocks capable of forming dimensionally controlled self-assembled crystalline fullerene nanomaterials at a liquid-liquid interface under ambient conditions of temperature and pressure [12,13,14,15,16,17,18,19,20,21,22]. These nanomaterials include 0D particles or spheres, 1D nanorods or nanotubes, 2D nanodisks or nanosheets, 3D cubes or superstructures of cubes with rods protruding from the cube surfaces, and gyroid type nano/micro cubes [12,13,14,15,16,17,18,19,20,21,22]. Carbon materials with hollow interiors and micro/mesoporous structures offer unique functionality and provide voids for the storage of a large volume of charge due to enhanced electrolyte ion diffusion through the nanospaces [23,24]. As a result, carbon materials with high surface areas and hierarchically porous architectures are in great demand for use in high energy storage electrical double-layer capacitors (EDLCs) or supercapacitors [25,26,27,28,29,30,31,32].

Recently, fullerenes (C_60_ or C_70_) have emerged as the novel *π*-electron rich carbon sources for the production of morphology-controlled high specific surface area nanoporous carbon materials, which have significant potential as electrode materials in state-of-the-art electrochemical energy storage supercapacitors [33,34,35,36]. Nanoporous carbon materials resulting from the high temperature carbonization of fullerene crystals exhibit excellent electrochemical supercapacitive performances. For instance, Shrestha and coworkers [33] have prepared high surface area nanoporous carbon rods (surface area: 1600 m^2^ g^−1^) and carbon tubes (surface area: 1650 m^2^ g^−1^) by the thermal annealing of crystalline fullerene (C_60_) nanorods and nanotubes at an extremely high temperature (2000 °C) in a vacuum. The resulting carbon materials exhibit highly graphitic and robust pore walls with a maximum of six graphene-like carbon layers having an interlayer spacing of 0.35 nm. The carbon tubes exhibited enhanced electrolyte ion diffusion, and the electrode attained a high specific capacitance of 145 F g^−1^ at a 5 mV s^−1^ scan rate due to a larger electrochemical surface and hollow interiors. The same workers have also demonstrated that the high temperature (2000 °C) carbonization of “Konpeito-like” 0D fullerene nano-objects results in the formation of highly micro/mesoporous carbon spheres containing sufficient void spaces for high energy storage applications (as an electrical double layer) [34]. Electrodes prepared from the carbon spheres derived from “Konpeito-like” 0D fullerene crystals exhibited a specific capacitance of 115 F g^−1^ at a current density of 1 A g^−1^ sustaining 45% of its capacitance at 10 A g^−1^ current density. Similarly, Bairi and coworkers [37] have demonstrated that mesoporous graphitic carbon microtubes with ordered conjugated *sp*^2^ carbon obtained by the thermal annealing of fullerene C_70_ microtubes at 2000 °C exhibit a large specific capacitance of 184 F g^−1^ at 0.5 A g^−1^ current density accompanied by an excellent rate performance of the electrode. Bairi and coworkers [10] extended their work on the thermal conversion of crystalline fullerene materials to porous carbon materials and consequently demonstrated hierarchical micro/mesoporous carbon cubes obtained by the thermal treatment of mesoporous fullerene C_70_ cubes at a relatively high temperature of 900 °C. Due to an enhanced surface area (642 m^2^ g^−1^) and an appropriate mesopore size (3.44 nm), the fullerene C_70_ crystal-derived carbon cubes performed well as the electrical double-layer capacitor electrode material in an aqueous electrolyte, and the electrode delivered a high specific capacitance of 205 F g^−1^ at 1 A g^−1^ without any significant loss of capacity after 10,000 consecutive charge/discharge cycles. Apart from pristine fullerene assemblies, mesoporous carbon materials have also been recently reported to be derived from C_60_/[6,6]-phenyl-C61-butyric acid methyl ester (PCBM) hybrid crystals [11]. Carbon materials obtained by the calcination of the C_60_/PCBM hybrid crystals showed good electrochemical performance due to their enhanced surface area. The electrode achieved a specific capacitance of 158 F g^−1^ at 1 A g^−1^ accompanied by 87.3% capacitance retention after 2000 cycles. These recent examples demonstrate the potential of fullerene-derived porous carbon materials in high-performance energy storage applications.

**Scheme 1:**
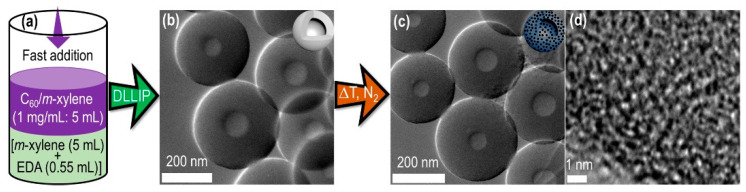
Preparation of self-assembled fullerene-ethylenediamine hollow spheres (FE-HS) and their direct conversion to hierarchically porous carbon spheres. (**a**) Schematic of the DLLIP method; (**b**) transmission electron microscopy (TEM) image of FE-HS with a representative model in the inset; (**c**) TEM image of the resulting hollow carbon spheres with a model structure in the inset; and (**d**) high-resolution TEM (HR-TEM) image of the hollow carbon spheres showing abundant micro/mesopores.

In this work, we disclose a simple method to fabricate fullerene-ethylenediamine (EDA) hollow spheres (FE-HS) of narrow size distribution (average size ca. 290 nm) under ambient conditions using the dynamic liquid-liquid interfacial precipitation (DLLIP) method. High temperature carbonization (700 to 1100 °C) of FE-HS results in the formation of hierarchically porous carbon nanospheres (Scheme 1). The resulting hollow carbon spheres exhibit excellent energy storage supercapacitor performance in an aqueous electrolyte due to their enhanced specific surface areas and well-developed micro- and mesoporosity of the carbon frameworks. Our results demonstrate a simple synthetic route to the scalable production of high surface-area hollow carbon spheres, which have enormous potential for electrochemical energy storage supercapacitor applications. 

## 2. Materials and Methods

### 2.1. Materials

Commercially available fullerene C_60_ (pC_60_: 99.5%) was obtained from BBS chemicals, Chimes Drive, Houston, TX, USA. Isopropyl alcohol (IPA: 99.7%), *m*-xylene (99.8%), and ethylenediamine (EDA: 99%) were purchased from Wako Chemicals Corporation, Tokyo, Japan. Acetylene black was obtained from Alfa Aesar, Ward Hill, Massachusetts, United States. Poly(vinylidene fluoride: PVDF) was purchased from Sigma Aldrich Co. Spruce Street, St. Louis, MO, USA. N-Methyl-2-pyrrolidone (NMP; 99.5%) was procured from Nacalai Tesque, Inc., Kyoto, Japan. All chemicals were used as received. 

### 2.2. Preparation of FE-HS and Carbonized FE-HS

A solution of pristine fullerene in *m*-xylene (1 mg mL^−1^) was prepared by dissolving pC_60_ (100 mg) in *m*-xylene (100 mL). A solution of EDA (0.55 mL) in *m*-xylene (5 mL) was prepared in a clean and dry glass bottle with a screw cap (13.5 mL) by vortex mixing and applying sonication for 30 min. A portion of the pC_60_ solution in *m*-xylene (5 mL) was then added into the EDA/*m*-xylene solution under ambient conditions, followed by thorough mixing on a vortex mixer for 10 s and incubation at 25 °C for 2 h. The resulting dark precipitate was separated by centrifugation (9500 rpm) for 10 min and washed three times with aliquots of IPA (5 mL). The resulting product, fullerene-EDA hollow spheres, was freeze-dried for 48 h and is referred to as FE-HS. The fullerene-EDA hollow spheres were carbonized at different temperatures (700, 900, and 1100 °C) in a tube furnace under an inert nitrogen gas atmosphere to obtain the hierarchically porous hollow carbon spheres (KOYO, Tokyo, Japan). During carbonization, the heating ramp, hold time, and nitrogen gas flow rate were set to 10 C min^−1^, 3 h, and 120 cc min^−1^, respectively. The resulting products were designated as FE-HS_700, FE-HS_900, and FE-HS_1100 according to the temperature of carbonization. The yield of the carbonized product was 67.2% (FE-HS_700), 56.3% (FE-HS_900), and 48.4% (FE-HS_1100).

### 2.3. Characterizations

FE-HS materials and the derived hierarchically porous hollow carbon spheres were characterized by Fourier-transform infrared (FTIR) in attenuated total reflection (ATR) mode using NICOLET iS20 (Thermo-Fisher Scientific, Waltham, MA, USA) spectroscopy, thermogravimetric analysis (TGA) on a STA 2500 (Regulus, NETZSCH, Wittelsbacherstraße, SELB, Germany), powder X-ray diffraction (XRD) using Rigaku X-ray diffractometer, RINT (Tokyo, Japan), and Raman scattering spectroscopy using a NRS-3100 (JASCO, Tokyo, Japan). Surface morphology was studied by scanning electron microscopy (SEM) using the S-4800 (Hitachi Co., Ltd. Tokyo, Japan) operated at 10 kV and 10 μA, and transmission electron microscopy (TEM) using the JEM2100F instrument (JEOL Tokyo Japan) operated at 200 kV. Nitrogen adsorption/desorption isotherms of the materials were recorded on an Autosorb-iQ2 (Quantachrome, Boynton Beach, FL, USA) for determination of the textural properties, including surface areas, pore volumes, and pore size distributions. The micropore size distribution profiles were obtained by the density functional theory (DFT) method, and the mesopore size distribution was estimated using the Barrett–Joyner–Halenda (BJH) model. Seaton and coworkers [38] have established the density functional theory (DFT), which describes the adsorption isotherm as the collection of independent, slit-shaped separate pores. The experimental adsorption isotherm, *N*(*p*), represents the average of all the pore sizes in the material and can be expressed as:

(1) $$N\left(P\right)=\int_{H_{min}}^{H_{max}}\rho \left(P,H\right)f\left(H\right)\;dH$$

where *H_min_* and *H_max_*, represent the smallest and largest pore widths, respectively. (*P*,*H*) relates to the mean density of adsorbed nitrogen gas in the pore with pore width of *H* at pressure *P*, and *f*(*H*) signifies the pore size distributions. Detail mathematical calculations and quantitative information of the DFT model can be found [39,40]. 

### 2.4. Electrochemical Measurements

The electrochemical testing of the material was conducted using a CH instruments electrochemical workstation ALS CHI 660. The half-cell study of the carbon material was performed in a three-electrode setup with working (carbon material), reference (Ag/AgCl), and counter (Pt coil) electrodes. The working electrode was made by casting a slurry of the carbon material onto 1.5 cm × 2 cm graphite sheets followed by drying at 80 °C in an oven. The slurry was prepared by mixing the active material, conductive additive (acetylene black), and binder poly(vinylidene fluoride) (PVDF) with N-methyl-2-pyrrolidone at a ratio of 80:10:10. Electrochemical measurements (cyclic voltammetry (CV), galvanostatic charge/discharge (GCD), and electrochemical impedance spectroscopy (EIS)) were performed on the electrodes in aqueous 1 M H_2_SO_4_ electrolyte. The best-performing material in the half cell was fabricated into a symmetric supercapacitor cell and tested for its electrochemical performance and cycling stability for 10,000 charge/discharge cycles. 

The specific capacitance was calculated from the GCD curves using Equation (2):

(2) $$C_{s}\left(F/g\right)=4\frac{idt}{dV}$$

where *i* is gravimetric current density (A), *dt* is difference in the discharge time (s), and *dV* is potential window (*V*). 

The energy density (*E*) and power density (*P*) of the symmetric cells were calculated using Equations (3) and (4), respectively.

(3) $$E\left(Wh/kg\right)=\frac{C_{s}V^{2}}{8\times 3.6}$$

(4) $$P\left(W/kg\right)=\frac{E*3600}{dt}$$

## 3. Results and Discussion

Homogeneous FE-HSs with a narrow size distribution were prepared using the DLLIP method under ambient conditions. In a typical synthesis, a solution of fullerene (C_60_) in *m*-xylene (1 mg mL^−1^: 5 mL) was added rapidly into a solution of ethylenediamine (EDA: 550 μL) in *m*-xylene (5 mL), followed by treatment using vortex mixing for 5 s. The resulting solution was incubated at 25 °C for 24 h and then subjected to centrifugation to isolate the solid product, followed by three times washing with isopropanol. Scanning electron microscopy (SEM) images reveal the uniform size distribution of the resulting FE-HSs (Figure S1), and the hollow interiors of the spheres can be observed by TEM (Figure S2a,b). FE-HS has a nonporous amorphous structure, as revealed by HR-TEM (Figure S2c,d). Size distributions of the outer and inner diameters were estimated using one hundred randomly selected FE-HSs. The resulting histograms are shown in Figure S2e (outer diameter) and Figure S2f (inner diameter). The outer diameter of FE-HSs is distributed in a narrow range from 220 to 360 nm with an average diameter of 290 nm, while the inner diameter is distributed in the range 20 to 110 nm with an average inner diameter of 65 nm. From the histograms, the average thickness of the sphere walls is ca. 225 nm. The uniform dimensions are an indication of the homogenous growth of the spheres at the liquid-liquid interface.

FE-HS formation is based on the amination reaction of fullerene with EDA [41,42,43,44]. A variety of dienes and nucleophiles react with fullerene resulting in the adducts. Notably, primary and secondary amines such as *n*-propylamine and ethylenediamine quickly react with fullerene C_60_ and give amino-added compounds. Sun and coworkers [45] have reported hollow spheres with a controllable wall thickness and a hollow size or cavity through the amination reactions of fullerene C_60_ with alkyl diamines, where the alkyl chain length of the alkyl diamines plays a crucial role and determines the cavity size. In the fullerene-EDA product, the EDA is cross-linked, which connects the fullerene C_60_. They proposed that one amino group is bonded to a C_60_ molecule, and the other amino group is bonded to another C_60_ molecule to form the cross-linked structure. Through the TEM imaging at different reaction times, they found that initially (10 min) solid spheres are without hollow interiors, and after 20 min, a hollow structure appears. The overall size of the spheres and nitrogen content increase with the reaction time and reach a maximum after 60 min [45]. Judging from these observations, a possible hollow structure formation mechanism is proposed as follows. In the early reaction stage (~10 min), EDA functionalized C_60_ through an amination reaction assembled to form solid aggregates. The fullerene C_60_, located on the outer surface of solid spheres, continues to react with EDA until the surface of the spheres is saturated. With the progress of the reaction, C_60_ located at the interior of spheres with unsaturated surfaces diffuses to the outer surface and reacts with EDA, leading to a hollow structure at the center of spheres. Thermogravimetric analysis (TGA) was performed to study the thermal decomposition behavior of the FE-HSs prior to carbonization. The TGA curve shows that the thermal decomposition process of the FE-HSs proceeds at different stages (Figure S3). The initial mass loss below 100 °C indicates the vaporization of moisture and/or alcohol trapped in the structure during the self-assembly of fullerenes. The second-stage mass loss at around 200 °C can be attributed to the removal of solvent *m*-xylene and any unreacted EDA. Note that the DLLIP results in the rapid formation of self-assembled fullerene-based nanomaterials and, as a result, some solvent molecules are trapped in the self-assembled nanomaterials. Mass loss in the range 300–750 °C can be attributed to the partial breakage of the crosslinked fullerene-EDA complex. Finally, the mass loss above 750 °C is caused by the disruption of fullerene C-C bonds, leading to the formation of amorphous carbons. Based on the thermal decomposition TGA curve, we performed carbonization at three different temperatures: 700, 900, and 1100 °C, and the resulting hollow carbon spheres are referred to as FE-HS_700, FE-HS_900, and FE-HS_1100, respectively.

**Figure 1:**
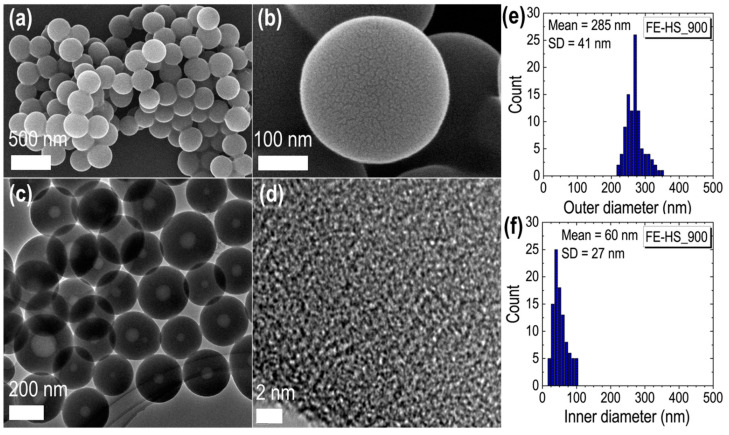
Electron microscopy observations of the hollow carbon spheres obtained by high temperature carbonization of FE-HS at 900 °C. (**a**,**b**) SEM images; (**c**) TEM image; (**d**) HR-TEM image; and (**e**,**f**) histograms of the outer diameter (**e**) and inner diameter (**f**) the FE-HS_900.

Figure 1 shows typical electron microscopy images (SEM: Figure 1a,b; TEM: Figure 1c; HR-TEM: Figure 1d) of the FE-HS_900 sample. Carbon spheres with a uniform size distribution can be observed in the SEM images (Figure 1a and Figure S4), while TEM images (Figure 1c and Figure S5a,b) reveal that the hollow space at the interior of the spheres remains intact following high-temperature carbonization. The dimensions of the hollow carbon spheres are similar to those of the starting material, FE-HS. From the histograms, the average outer and inner diameters are ca. 285 and 60 nm, respectively (Figure 1e,f), indicating that the carbon spheres have an average wall thickness similar to that of the starting FE-HS at 225 nm. However, in contrast to the starting material, a well-developed micro/mesoporous amorphous carbon structure can be observed in the FE-HS_900 sample (see HR-TEM images (Figure 1d and Figure S5c,d)). Hierarchical micro/mesopores are formed as a result of the thermal decomposition of fullerene and crosslinked fullerene-EDA molecules at high temperatures [45]. Hollow amorphous carbon spheres of similar dimensions can be observed in samples following carbonization of FE-HS at 700 °C (FE-HS_700: Figures S6 and S7) and 1100 °C (FE-HS_1100: Figures S8 and S9). FE-HS_700 and FE-HS_1100 samples also contain abundant micro/mesopores, making them suitable for high energy storage applications.

**Figure 2:**
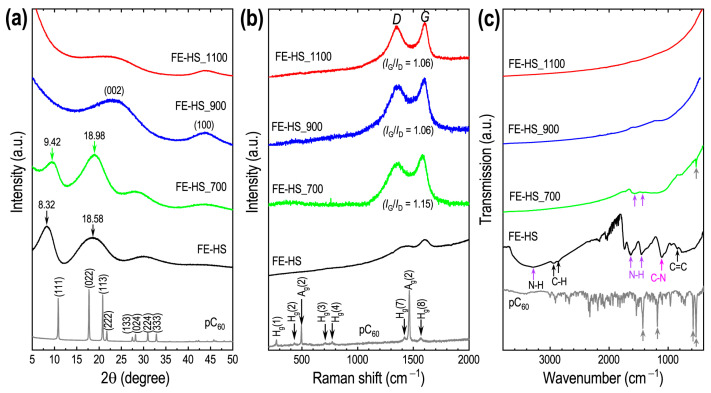
(**a**) pXRD patterns of pristine C_60_ (pC_60_), FE-HS, FE-HS_700, FE-HS_900; and the corresponding Raman scattering spectra (**b**), and FTIR spectra (**c**).

Figure 2 shows powder X-ray diffraction (pXRD) patterns, Raman scattering, and Fourier-transform infrared spectroscopy (measured using the attenuated total reflection technique, ATR) data for the FE-HS starting material and the thermally annealed samples: FE-HS_700, FE-HS_900, and FE-HS_1100.

Considering the pXRD patterns (Figure 2a), while pristine fullerene C_60_ is a crystalline material with face-centered cubic (*fcc*) structure, the self-assembled fullerene-EDA hollow spheres exhibit an amorphous structure. Two major broad diffraction peaks were observed at the diffraction angles (2θ) of 8.32° and 18.58°, and the lack of any crystalline peaks corresponding to pC_60_ indicates that FE-EDA has an amorphous structure with an interlayer *d*-spacing of ca. 1.602 nm. Following high temperature treatment, the smallest angle diffraction peak shifts slightly to a wider angle, indicating *d*-spacing of 0.938 nm in the FE-HS_700 sample. These diffraction peaks are strongly attenuated with increasing temperature, and new broad peaks emerged at around 24° and 43°, corresponding to the (002) and (100) planes of disordered graphite-like amorphous carbon (Figure 2a) [46]. The amorphous structure of the heat-treated samples was further confirmed by studying Raman spectra (Figure 2b). The pC_60_ crystalline material exhibits several Raman vibration bands (six H_g_ vibrations and two A_g_ vibration bands) [47]. However, molecular rotations of the fullerene molecules are restricted in the self-assembled FE-HS system due to crosslinking of the fullerene and EDA molecules; therefore, most of the Raman bands corresponding to the crystalline fullerene are absent in the spectrum of the FE-HS sample (Figure 2b). It should be noted that EDA reacts with fullerene, forming a C_60_-EDA complex, which forms amorphous hollow spherical assemblies [45]. High temperature heat-treatment transforms the C_60_-EDA spheres into amorphous carbon materials due to the random breakage of the C_60_-EDA bonds and also due to breakage of the fullerene C-C bonds. As a result, *D* and *G* bands corresponding to the defective, graphitic structure of amorphous carbon [48] are observed in the Raman spectra of the high temperature carbonized samples FE-HS_700, FE-HS_900, and FE-HS_1100 (Figure 2b). The intensity ratio of the *G* and *D* bands lies in the range 1.06 to 1.15, typical of amorphous carbon with a partial graphic structure. The FTIR-ATR spectrum of FE-HS differs from that of crystalline pC_60_. FTIR bands of the FE-HS include a broad N-H (str.) band in the range 3100–3500 cm^−1^, a N-H (def.) vibration at 1635 and 1450 cm^−1^, and a C-N (str.) band at 1103 cm^−1^ due to the presence of EDA as a C_60_-EDA complex structure [22,49]. Following heat treatment (thermal annealing), the intensities of FTIR bands corresponding to N-H (def.) as indicated by purple arrows, C-N (str.), and bands corresponding to pristine C_60_ (as indicated by gray arrow) decrease significantly for the FE-HS_700 sample (Figure 2c), followed by further decreases in the spectra of the FE-HS_900 and FE-HS_1100 samples due to the high temperature carbonization process.

**Figure 3:**
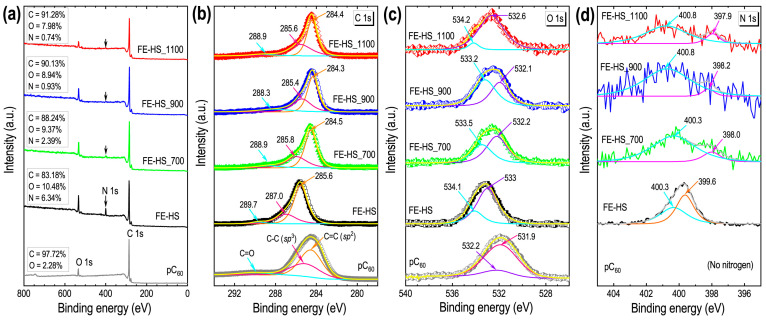
X-ray photoelectron spectra of the materials. (**a**) XPS survey spectra of pC_60_, FE-HS, FE-HS_700, FE-HS_900, and FE-HS_1100; (**b**) XPS C 1s core level spectra with the deconvoluted peaks; (**c**) XPS O 1s spectra with the deconvoluted peaks; and (**d**) XPS N 1s spectra with the deconvoluted peaks.

The surface chemistry of FE-HS and the hierarchically porous hollow carbon spheres derived from it were studied by X-ray photoelectron spectroscopy (XPS) (Figure 3).

The survey XPS spectra (Figure 3a) indicate that the surfaces of the prepared carbon samples are partially oxidized. Three major XPS peaks are observed at 284, 400, and 532 eV in the FE-HS due, respectively, to carbon, nitrogen, and oxygen, which are the main components at the materials’ surfaces. Nitrogen in the samples originates from EDA present in the system. As expected, pC_60_ contains only carbon and oxygen (the latter due to partial oxidation) as its surface components. Increases in the carbonization temperature decreases the intensity of the XPS peaks corresponding to oxygen and nitrogen (Figure 3a), indicating lower contents of those elements in the carbonized hollow carbon spheres. The nitrogen content in the as-prepared FE-EDA is ca. 6.34 atom%, which decreases according to temperature after carbonization, finally reaching ca. 0.74 atom% in the FE-HS_1100 sample. The deconvoluted XPS core level high resolution C 1s spectrum indicates that pC_60_ consists largely of C=C (*sp*^2^), C-C (*sp*^3^), and C=O bonding states of carbon (Figure 3b) [50]. While the FE-HS sample contains an additional C-N bonding state of carbon due to the presence of EDA, the carbon hollow spheres (FE-HS_700, FE-HS_900, and FE-HS_1100) have mostly C=C, C-C, and C=O bonding states of carbon. The O 1s XPS core level-spectra were deconvoluted to two peaks (with a partial peak shift) corresponding to the C-OH and C-O-C bonding states (Figure 3c). The deconvoluted N 1s high resolution XPS spectrum of FE-HS shows that nitrogen is present both in NH_2_ (399.6 eV) and positively charged nitrogen (400.3 eV) states (Figure 3d). In the high-temperature carbonized samples (FE-HS_900 and FE-HS_1100), nitrogen is largely present in a graphitic (400.8 eV) state, which is anticipated to contribute to improving the conductivity of these carbon materials, in turn enhancing their electrochemical energy storage properties.

**Figure 4:**
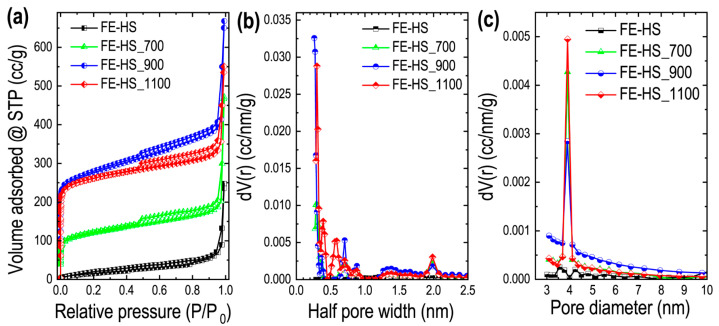
(**a**) Nitrogen adsorption isotherms FE-HS, FE-HS_700, FE-HS_900, and FE-HS_1100; (**b**) corresponding pore size distribution profile obtained by the density functional theory (DFT) method; and (**c**) pore size distribution and the Barrett–Joyner–Halenda (BJH) model.

Nitrogen adsorption/desorption isotherms were collected to determine specific surface areas, pore volumes, and pore size distributions of FE-HS and its derived porous hollow carbon spheres (FE-HS_700, FE-HS_900, and FE-HS_1100) (Figure 4). The nitrogen sorption isotherm of the FE-HS starting material reveals a low nitrogen intake (adsorption) due to a lack of porosity, which was also evident from its HR-TEM images (Figure S2c,d). It exhibits a Type III isotherm, which typically corresponds to nonporous materials. Nitrogen adsorption increases drastically in the thermally annealed samples, indicating the large porosity of those materials, with nitrogen uptake being dependent on the carbonization temperature. Nitrogen uptake increases with higher carbonization temperatures up to 900 °C, then decreases slightly in the 1100 °C sample. All the carbonized samples display Type-I/Type-IV, mixed type sorption isotherms (Figure 4a), suggesting the formation of hierarchically micro/mesoporous architectures [51,52,53,54]. Large nitrogen adsorption at lower relative pressures (P/P_0_ < 0.05) can be attributed to micropore filling, while gradual nitrogen adsorption at higher relative pressures with a hysteresis loop can be attributed to capillary condensation taking place in mesopores. Careful observation reveals that nitrogen adsorption at the lower relative pressures (micropores) increases with increasing carbonization temperature up to 900 °C, then decreases slightly, suggesting that the development of microporosity occurs during high temperature carbonization increasing up to 900 °C. Micropore coalescence results in an increasing population of mesopores during high-temperature carbonization at 1100 °C. Thus, microporosity decreases while mesoporosity increases in the FE-HS_1100 sample. A similar trend has been observed in chemically activated nanoporous carbon materials obtained from biomass.

Pore size distributions obtained from analyses of the isotherms by the DFT method (Figure 4b) and BJH model (Figure 4c) contain obvious peaks due to micropores (Figure 4b) and mesopores (Figure 4c) confirming the formation of hierarchical pore architectures of the hollow carbon spheres. The surface textural parameters summarized in Table 1 reveal the effects of carbonization temperature on the surface area and porosity of the samples. Both FE-HS_900 and FE-HS_1100 samples offer excellent specific surface areas and hierarchical micro- and mesopore architectures with appropriate pore size distributions for high energy storage supercapacitors.

**Figure 5:**
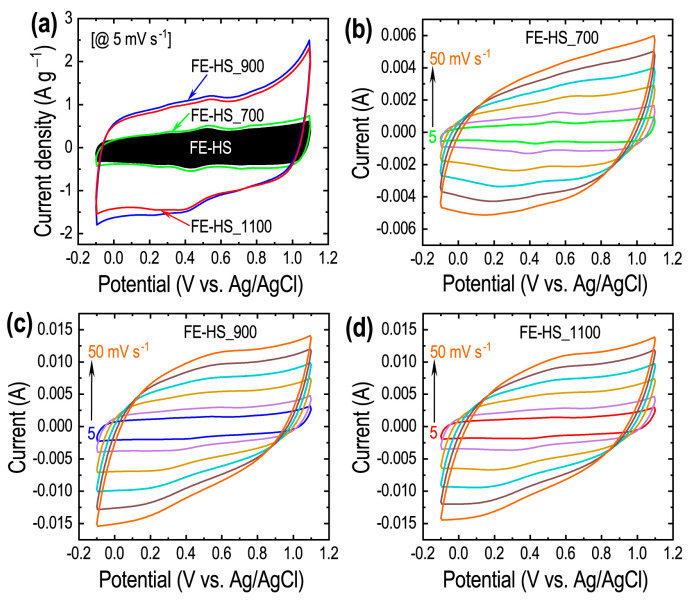
(**a**) CV curves of FE-HS, FE-HS_700, FE-HS_900, and FE-HS_1100 recorded at a scan rate of 5 mV s^−1^ in the three-electrode cell setup; (**b**) CV curves vs. scan rates for FE-HS_700; (**c**) CV curves vs. scan rates for FE-HS_900; (**d**) CV curves vs. scan rates for FE-HS_1100.

Based on the high surface areas, large porosities, and well-defined appropriate micro- and mesopore size distributions, the electrochemical energy storage supercapacitance performances of the FE-HS-derived hollow carbon spheres were studied by using CV, GCD, and EIS in an aqueous electrolyte (1 M H_2_SO_4_) solution. Figure 5a compares the CV profiles of the as-prepared FE-HS with the carbonized samples FE-HS_700, FE-HS_900, and FE-HS_1100 at a potential difference of 1.2 V. All the samples exhibit capacitive charge storage (EDLC behavior), as indicated by the quasi-rectangular CV curves [55,56,57]. A minor redox peak at 0.5 V (oxidation) and 0.4 V (reduction) with insignificant intensity is due to the surface functionalities (oxygen and nitrogen). As expected from the surface textural properties, the total integral current collected in the FE-HS_900 sample is highest among these samples, suggesting micropore-driven enhanced energy storage performance (Table 1). The current collection for FE-HS_1100 is comparable to that for FE-HS_900. FE-HS has the lowest integral current collection (Figure 5a), indicating its poor energy capacity due to a lack of porosity. Although the specific surface area of the FE-HS_700 sample is significantly larger than that of FE-HS, the total current in the CV profile is only slightly greater. This can be attributed to the low conductivity of FE-HS_700 caused by the presence of some fullerene-EDA complex (Figure 2a) and its lower state of carbonization. The CV profiles recorded at different scan rates (5 to 50 mV s^−1^) sustain a quasi-rectangular shape (Figure 5b–d and Figure S10), suggesting fast electrolyte ion diffusion to the electrode surface supported by the hierarchical micro/mesopore architecture.

**Figure 6:**
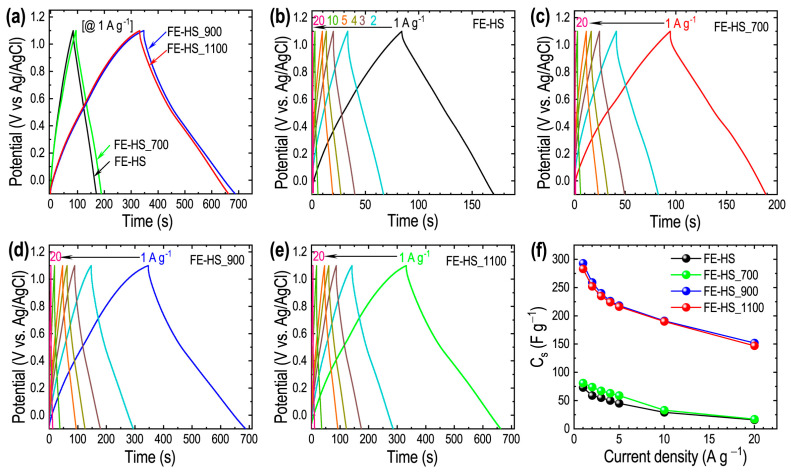
(**a**) GCD profiles of FE-HS, FE-HS_700, FE-HS_900, and FE-HS_1100 measured at a current density of 1 A g^−1^; GCD curves at different current densities (1 to 20 A g^−1^) for (**b**) FE-HS; (**c**) FE-HS_700; (**d**) FE-HS_900; and (**e**) FE-HS_1100; and (**f**) calculated specific capacitance from the GCD curves against current density for all the samples.

The supercapacitance energy storage properties studied by GCD measurements are summarized in Figure 6. Figure 6a compares the GCD profiles of the electrodes at a fixed current density (1 A g^−1^). EDLC behavior with well-balanced charge storage is evident from the triangular shape of the charge-discharge curves [58,59]. Note that the FE-HS_900 sample with the optimal surface area shows the longest discharge time, inferring the highest energy storage capacity of the electrode. The discharge time of the FE-HS_1100 electrode is comparable.

Based on the charge discharge curve data, FE-HS, and FE-HS_700 have low specific capacitances of 73 and 81 F g^−1^, respectively, while FE-HS_900 and FE-HS_1100 exhibit much higher values of 293 and 283 F g^−1^ at 1 A g^−1^, respectively. These values are associated with their large specific surface areas and a decreasing *I*_G_/*I*_D_ ratio found by using Raman spectroscopy. The increase in capacitance is largely a result of increases in disorder upon thermal treatment at high temperatures (evident from the Raman spectra), which increases the surface area, leading in turn to increased charge storage. The GCD profiles of the electrodes recorded at different current densities from 1 to 20 A g^−1^ (Figure 6b–e) shows the retention of a quasi-rectangular shape of the GCD curve even at a higher current density of 20 A g^−1^, suggesting a fast electrolyte ion transport to the electrode surface. The rate performances of the FE-HS_900 and FE-HS_1100 samples are also good, providing good specific capacitances of 152 F g^−1^ (51.8%) and 147 F g^−1^ (51.9%), respectively, at a current rate of 20 A g^−1^ (Figure 6f), demonstrating the significant potential of FE-HS-derived hierarchical hollow porous carbon materials for high-rate performance supercapacitor applications. Our materials’ overall energy storage performance is comparable to other porous carbons or related materials (Supplementary materials, Table S1).

**Figure 7:**
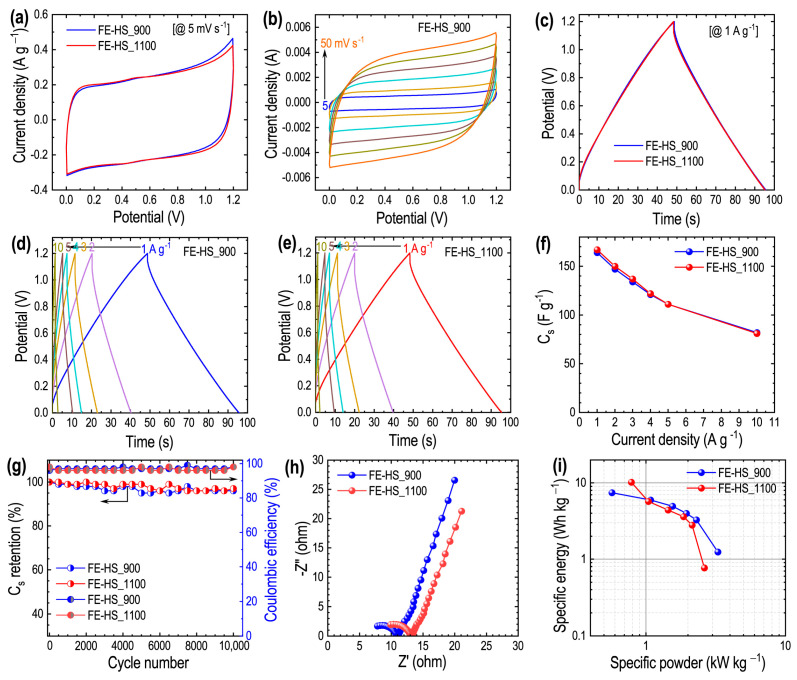
Electrochemical performance of the assembled symmetric supercapacitors. (**a**) Comparison of the CV curves recorded at a fixed scan rate of 5 mV s^−1^ for FE-HS_900 and FE-HS_1100; (**b**) CV curves vs. scan rate for FE-HS_900; (**c**) GCD curves of FE-HS_900 and FE-HS_1100 at 1 A g^−1^; GCD profiles vs. current density for (**d**) FE-HS_900; and (**e**) FE-HS_1100; (**f**) calculated specific capacitance vs. current density; (**g**) cycle performance with coulombic efficiency; (**h**) Nyquist plots for FE-HS_900 and FE-HS_1100; and (**i**) Ragone plot of the FE-HS_900 and FE-HS_1100 supercapacitors.

The high-performing electrode materials (FE-HS_900, and FE-HS_1100) were considered for the two-electrode system studies using a symmetrical configuration. The electrochemical supercapacitance performance of the resulting symmetric supercapacitors is shown in Figure 7.

Figure 7a shows the CV profiles of both systems (FE-HS_900 and FE-HS_1100) at a fixed scan rate of 5 mV s^−1^. The EDLC charge storage behavior of both can be judged from the rectangular shapes of the CV curves. The total integral current in the CV curves is comparable, suggesting that FE-HS_900 and FE-HS_1100 samples have similar energy storage capacities without a significant difference in the specific capacitance values. The CV curves vs. scan rate (5 to 50 mV s^−1^) profiles are shown in Figure 7b (FE-HS_900) and Figure S11 (FE-HS_1100). The rectangular shape of the CV curves is sustained at the high scan rate of 50 mV s^−1^, indicating fast electrolyte ion diffusion due to the presence of mesoporous channels with interconnected pore structures. The EDLC behavior of the cell is further complemented by the GCD profiles (Figure 7c–e). Both systems have similar discharge times, indicating similar energy storage capacities (Figure 7c). The specific capacitances of the symmetric cells constructed using FE-HS_900 and FE-HS_1100 are, respectively, 164 F g^−1^ and 167 F g^−1^ at a current density of 1 A g^−1^ (Figure 7f) and are accompanied by relatively good rate performances of about 50% capacitance retention at 10 A g^−1^. Similarities in the performances of these materials are attributable to the comparable physical properties of the samples having similar pore sizes, degrees of disorder, and elemental compositions. Both samples exhibit excellent cycling stability with 96% capacitance retention without significant losses in columbic efficiency (98%) after 10,000 consecutive charge/discharge cycles (Figure 7g). Nyquist plots obtained from the EIS measurements (Figure 7h) consist of semi-circles in the high frequency region with a ~45° slope in the low frequency region, suggesting charge transfer resistance and Warburg impedance, which are commonly observed in oxygen and nitrogen-containing carbon materials [60,61,62]. The similarity in diameter of the semi-circles suggests comparable charge-transfer resistance in both systems. The energy performance of the assembled symmetric supercapacitors is summarized in the Ragone plot (Figure 7i). The symmetric devices delivered a specific energy and specific power of 7.44 Wh kg^−1^ and 0.57 kW kg^−1^ (FE-HS_900) and 10.2 Wh kg^−1^ and 0.78 kW kg^−1^ (FE-HS_1100).

Our present results and the recent examples of the energy storage performance of porous carbon-based materials infer that several key parameters contribute to improving the supercapacitance performance of the electrode materials or assembled supercapacitors. For example, the electrode material with high porosity, well-defined pore size, hierarchically porous structures consisting of a micro/mesopore architecture, interconnected pores, heteroatom doping, such as nitrogen or sulfur, a framework structure (graphitic), high conductivity, and suitable wetting properties would show outstanding energy performances, including high energy density, high specific capacitance, excellent rate performance, and a long cycle life without much loss of capacity. Nevertheless, it is challenging to produce such unique carbon materials with all of these properties. Production of novel heteroatom-doped hierarchically porous carbon nanohybrids with efficient pseudocapacitive materials would be one way to optimize the energy performance of the hybrid supercapacitors. The electrochemical reactions, electrostatic interaction, and synergistic effects from the EDLC and pseudocapacitive materials are anticipated to result in the supercapacitors’ incredibly high energy storage performance.

## 4. Conclusions

In summary, self-assembled fullerene-ethylenediamine hollow spheres (FE-HS) were synthesized by the dynamic liquid-liquid interfacial precipitation method. It is a simple, scalable method performed under ambient conditions. Homogeneous FE-HSs have a narrow size distribution and were converted directly into porous hollow carbon spheres by high-temperature carbonization in an inert nitrogen gas atmosphere. The resulting carbon materials retained the morphology, dimensions, and internal hollow structure of the starting materials and exhibit high specific surface areas and large pore volumes due to the formation of hierarchical micro- and mesopore architectures with interconnected pores. The optimum surface textural properties were obtained in the material, which had been carbonized at 900 °C. The specific surface area and pore volume were ca. 1616 m^2^ g^−1^, and 1.295 cm^3^ g^−1^, respectively, far superior to the commercially available activated carbon materials. A supercapacitor electrode prepared from the sample with the optimum textural properties showed excellent electrochemical supercapacitance performance in an aqueous 1 M sulfuric acid (H_2_SO_4_) solution in a three-electrode system. A high specific capacitance of 293 F g^−1^ was achieved at 1 A g^−1^ current density, accompanied by a high-rate performance of 51.8% capacitance retention at a large current density of 20 A g^−1^. Additionally, in the two-electrode cell setup, the assembled symmetric cell delivered a high specific capacitance of 164 F g^−1^ at 1 A g^−1^ with 50% capacitance retention at 10 A g^−1^ accompanied by 96% cycle life and 98% coulombic efficiency after 10,000 consecutive charge/discharge cycles. Our results reveal the significant potential of the self-assembled fullerene-ethylenediamine hollow sphere assemblies for the scalable production of high surface area hierarchical nanoporous carbon materials, which can be used to enhance the energy storage performance of supercapacitors.

## Figures and Tables

**Table 1:** Porosity properties of the starting material FE-HS, and the derived porous carbon materials obtained by the carbonization of FE-HS at different temperatures (700–1100 °C) ^1^.

Sample	*SSA* (m^2^ g^−1^)	*S*_micro_ (m^2^ g^−1^)	*S*_meso_ (m^2^ g^−1^)	*V*_p_ (cm^3^ g^−1^)	*V*_micro_ (cm^3^ g^−1^)	*W*_p_ (nm)	*D*_p_ (nm)
FE-HS	94.2	42.4	51.8	0.530	0.154	---	---
FE-HS_700	612.9	499.4	113.5	0.925	0.350	0.299	3.89
FE-HS_900	1616.5	1504.3	112.2	1.295	0.655	0.262	3.89
FE-HS_1100	1439.3	1264.2	175.1	1.346	0.630	0.274	3.90

^1^ *SSA* = specific surface area, *S*_micro_ = surface area contributed from micropores, *S*_meso_ = surface area of mesopores, *V*_p_ = total pore volume, *V*_micro_ = micropore volume, *W*_p_ = micropore size (average half pore width), *D*_p_ = mesopore size (average pore diameter).

## Data Availability

The data presented in this study are available on request from the corresponding authors.

## Supplementary Materials

The following supporting information can be downloaded at: https://www.mdpi.com/article/10.3390/nano13050946/s1, Figure S1: SEM images of FE-HS; Figure S2: TEM and HR-TEM images of FE-HS with the histograms of the outer and inner diameter distributions; Figure S3: TGA curve of the FE-HS; Figure S4: Additional SEM images of FE-HS_900; Figure S5: Additional TEM and HR-TEM images of FE-HS_900; Figure S6: SEM images of FE-HS_700; Figure S7: TEM and HR-TEM images of FE-HS_700 with the histograms of outer and inner diameter distributions; Figure S8: SEM images of FE-HS_1100; Figure S9: TEM and HR-TEM images of FE-HS_1100 with the histograms of the outer and inner diameter distributions; Figure S10: CV vs. scan rate profiles of the FE-HS system in the three-electrode cell setup; Figure S11: CV curves vs. scan rate of the symmetric cell-prepared FE-HS_1100 sample; Table S1: Comparison of the specific capacitance of similar carbon materials. References [63,64,65,66,67,68,69,70,71,72,73,74,75,76] are cited in the supplementary materials.
