# Supplementary material for: Nanoporous Hollow Carbon Spheres Derived from Fullerene Assembly as Electrode Materials for High-Performance Supercapacitors
Source: Nanomaterials (Basel). 2023 Mar 5;13(5):946. doi: 10.3390/nano13050946 (PMC10005309; doi:10.3390/nano13050946)
Supplement: Supplementary file 1 [file nanomaterials-13-00946-s001.zip › nanomaterials-2233786-supplementary.pdf]

nanomaterials

Electronic Supporting Information

Nanoporous Hollow Carbon Spheres Derived from Fullerene Assembly as Electrode Materials for High-Performance Supercapacitors

Lok Kumar Shrestha [1,2,*], Zexuan Wei [1,3] Gokulnath Subramaniam [4,5], Rekha Goswami Shrestha [1], Ravi Singh [2], Marappan Sathish [4,5], Renzhi Ma [1], Jonathan P. Hill [1], Junji Nakamura [6], and Katsuhiko Ariga [1,3,*]

[1] International Center for Materials Nanoarchitectonics (WPI-MANA), National Institute for Materials Science (NIMS), 1-1 Namiki, Tsukuba, Ibaraki 305-0044, Japan
[2] Department of Materials Science, Faculty of Pure and Applied Sciences, University of Tsukuba 1-1-1, Tennodai, Tsukuba, Ibaraki 305-8573, Japan
[3] Department of Advanced Materials Science, Graduate School of Frontier Sciences, The University of Tokyo, 5-1-5 Kashiwanoha, Kashiwa, Chiba 277-8561, Japan
[4] Electrochemical Power Sources Division, CSIR-Central Electrochemical Research Institute, Karaikudi 630003, Tamilnadu, India
[5] Academy of Scientific and Innovative Research (AcSIR), Ghaziabad- 201002, India
[6] Mitsui Chemicals, Inc., Carbon Neutral Research Center (MCI–CNRC), International Institute for Carbon-Neutral Energy Research (I2CNER), Kyushu University, 744 Motooka, Nishi-ku, Fukuoka-shi, Fukuoka 819-0395, Japan
* Correspondence: SHRESTHA.Lokkumar@nims.go.jp (L.K.S.); ARIGA.Katsuhiko@nims.go.jp (K.A.)

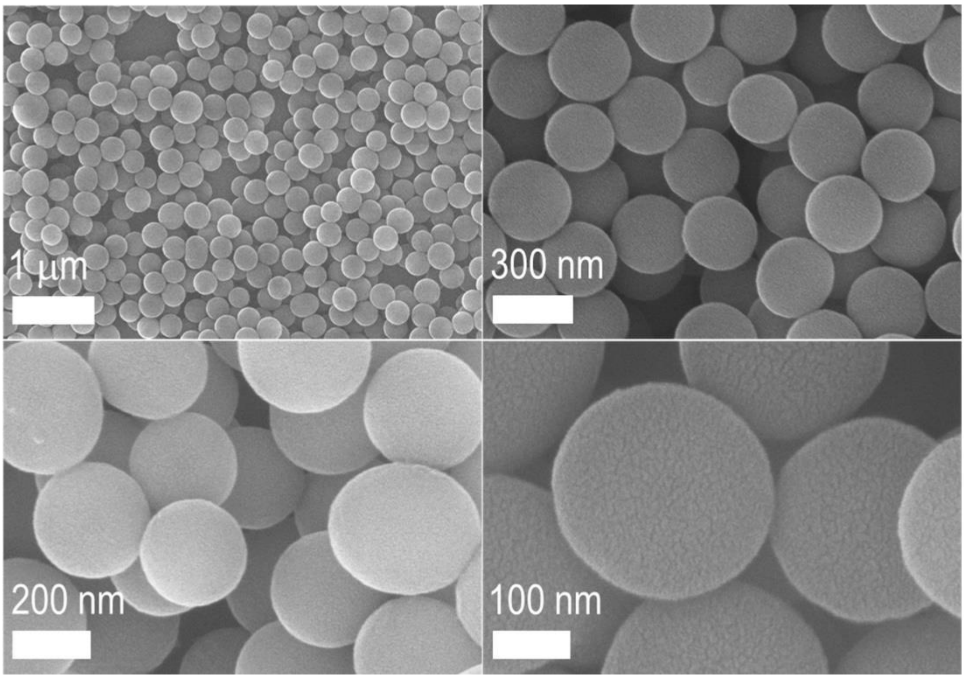

Figure S1: SEM images of FE-HS.

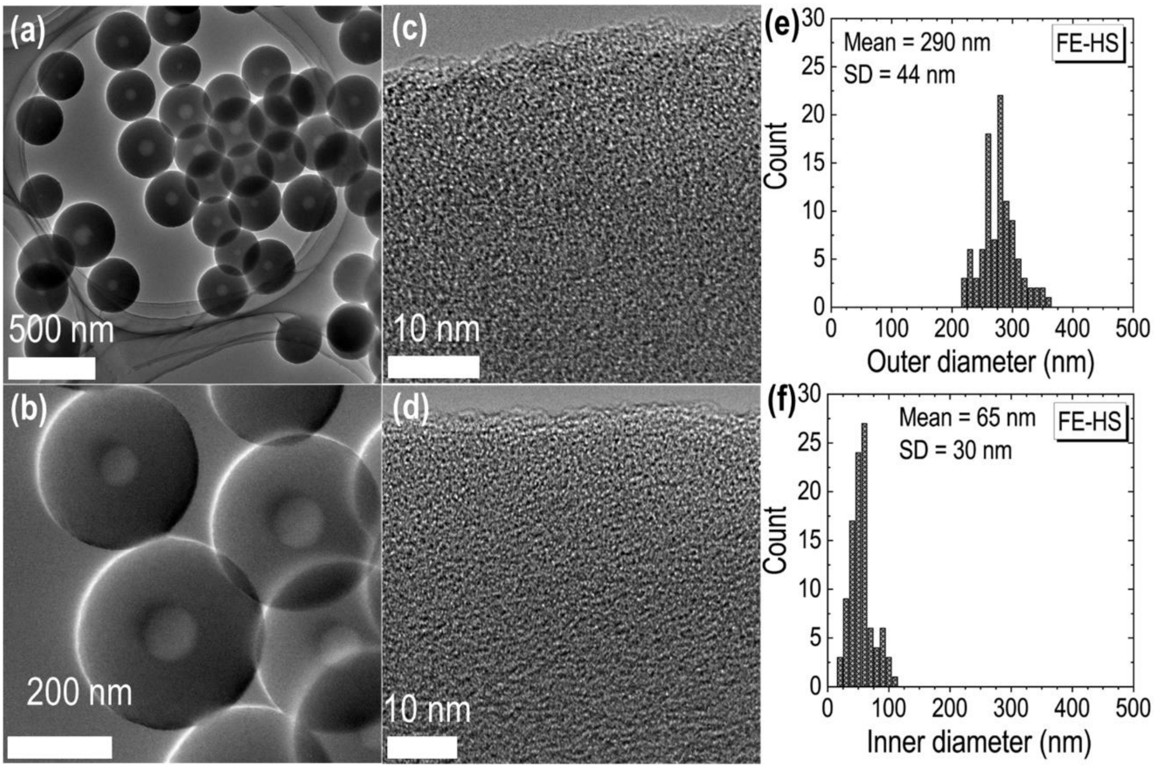

Figure S2: (**a-b**) TEM images; (**c-d**) HR-TEM images; (**e**) histogram of the outer diameter distribution; and (**f**) histogram of the inner diameter distribution.

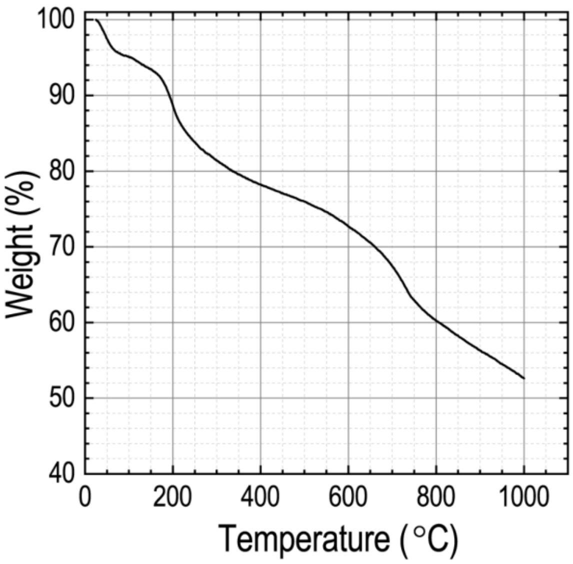

Figure S3: TGA curve of FE-HS.

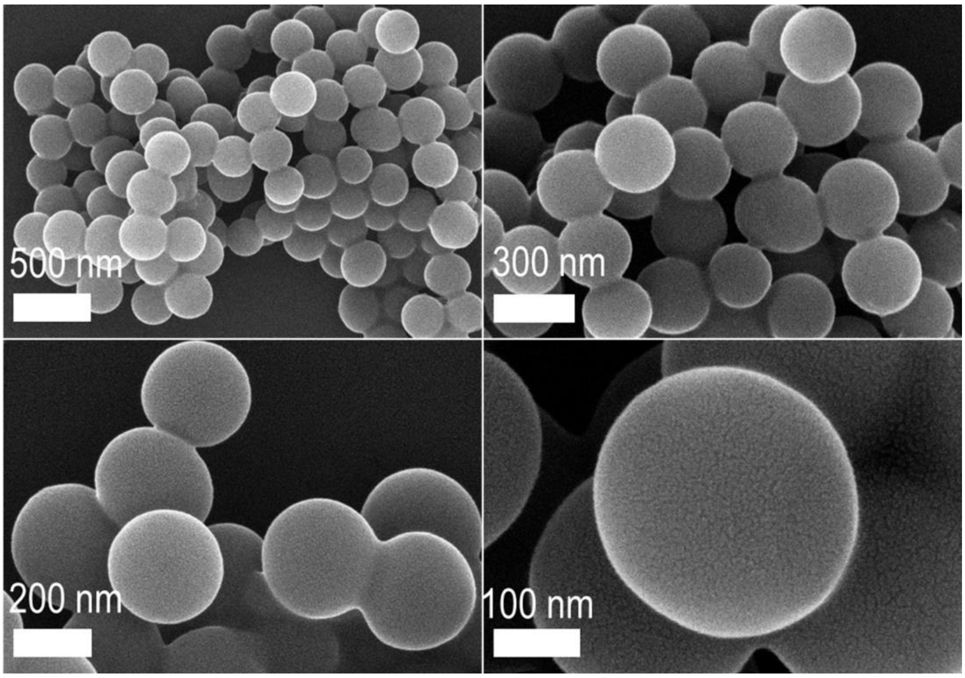

Figure S4: Additional SEM images of FE-HS_900.

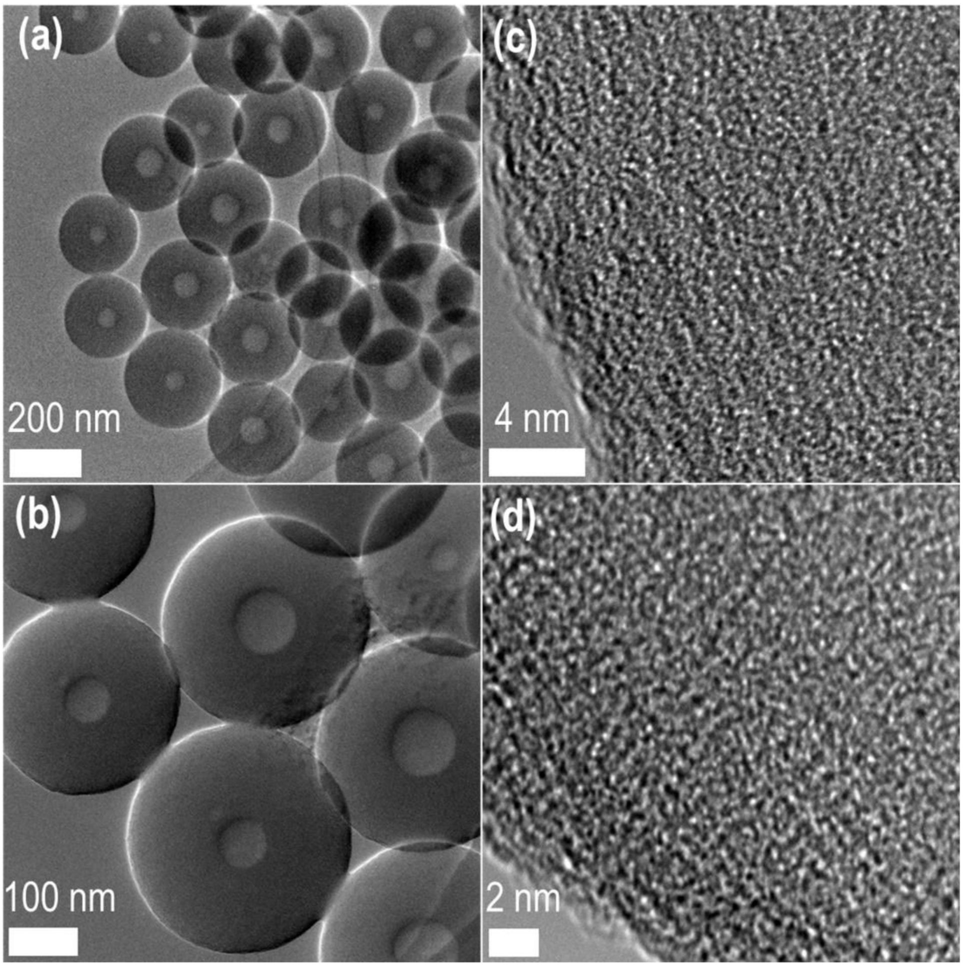

Figure S5: (**a-b**) Additional TEM images of FE-HS_900; and (**c-d**) corresponding additional HR-TEM images.

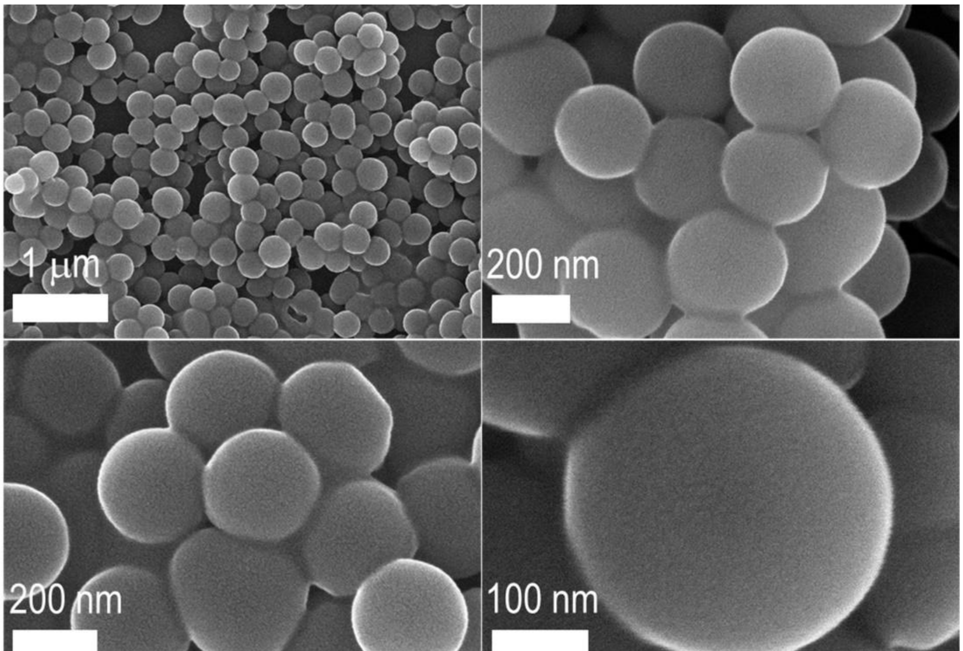

Figure S6: SEM images of FE-HS_700.

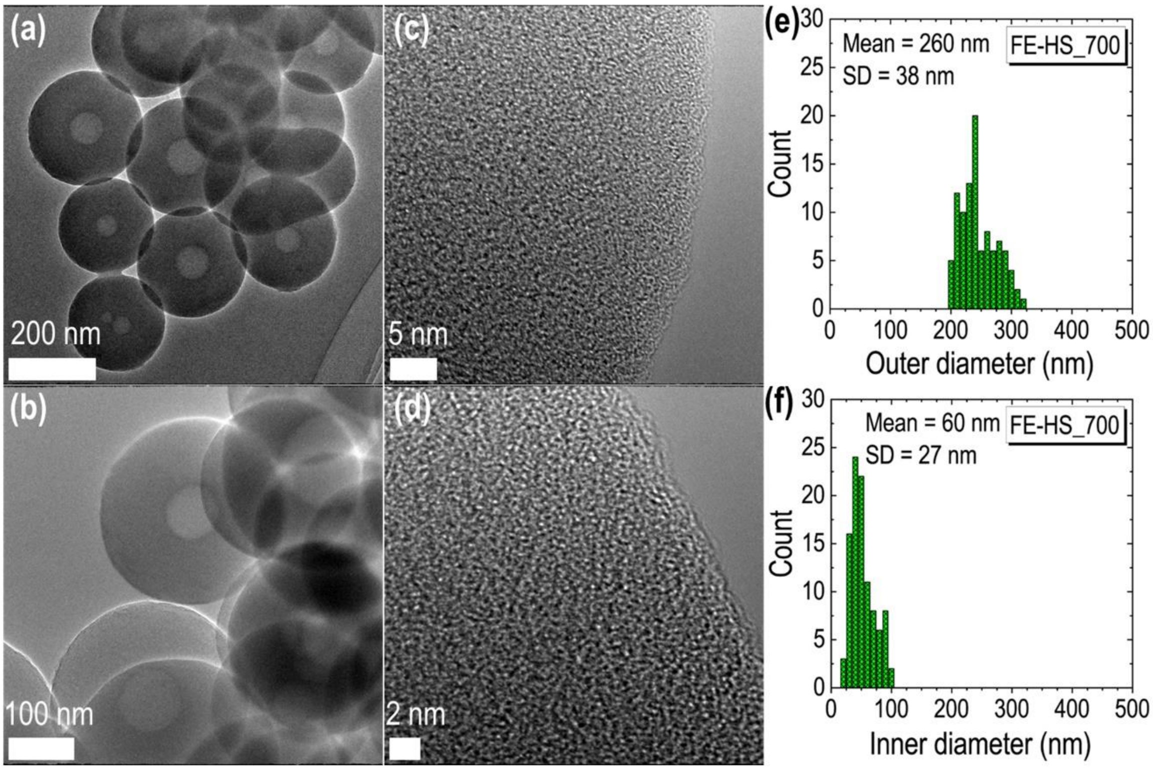

Figure S7: (**a-b**) TEM images; (**c-d**) HR-TEM images; and (**e-f**) histograms of the outer diameter (**e**) and inner diameter distributions (**f**) of EF-HS_700.

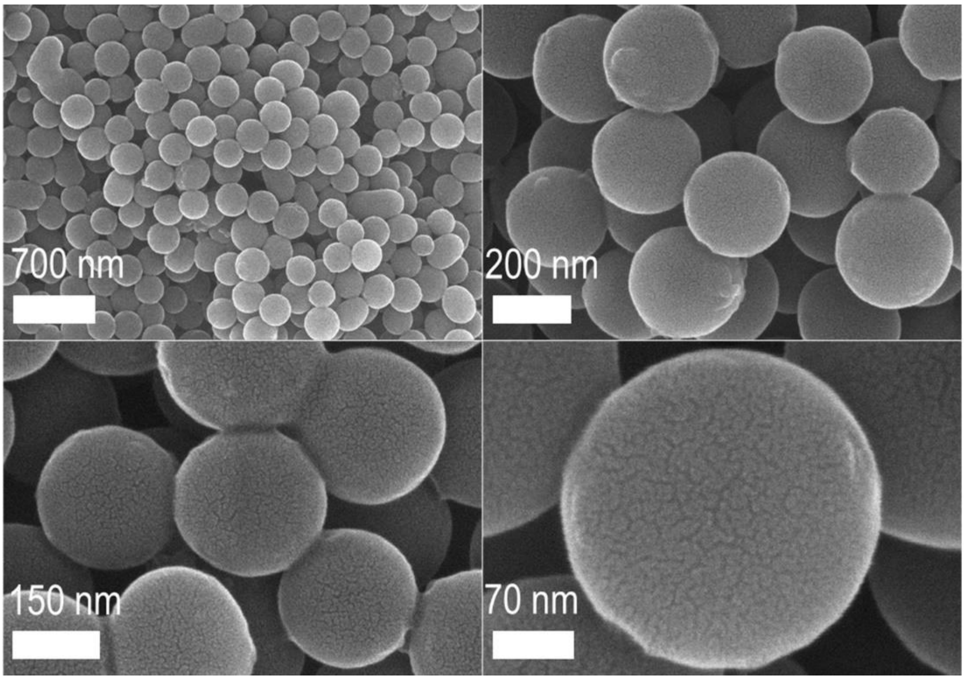

Figure S8: SEM images of FE-HS_1100.

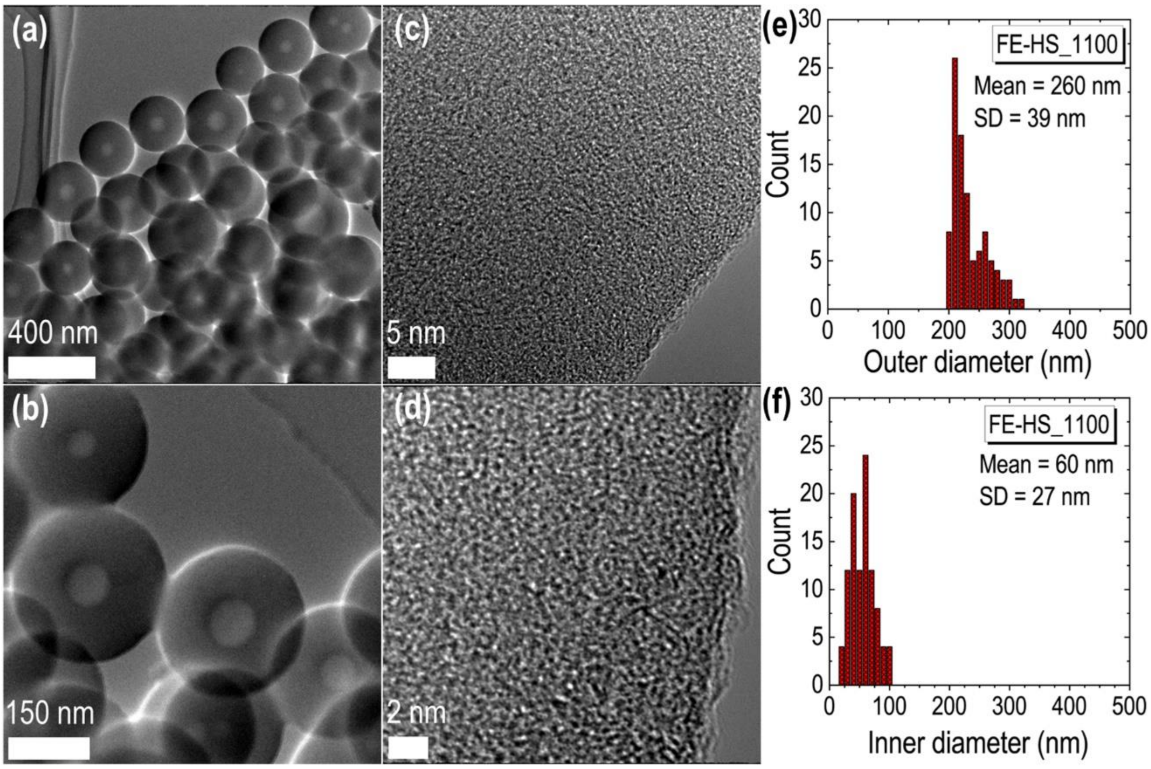

Figure S9: (**a-b**) TEM images; (**c-d**) HR-TEM images; and (**e-f**) histograms of the outer diameter (**e**) and inner diameter distributions (**f**) of EF-HS_1100.

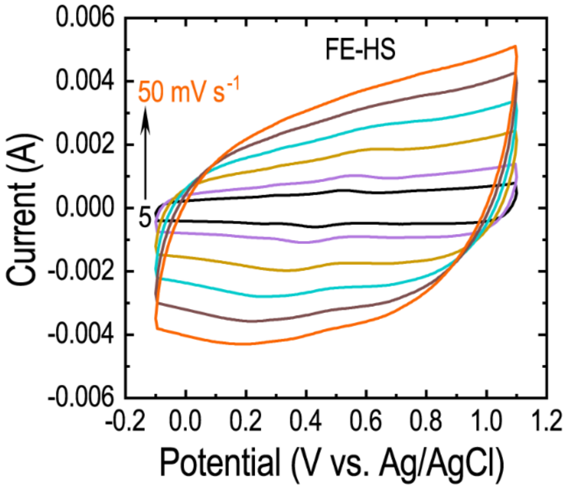

Figure S10: CV *vs*. scan rate profiles of the as-prepared FE-HS sample in a three-electrode cell setup.

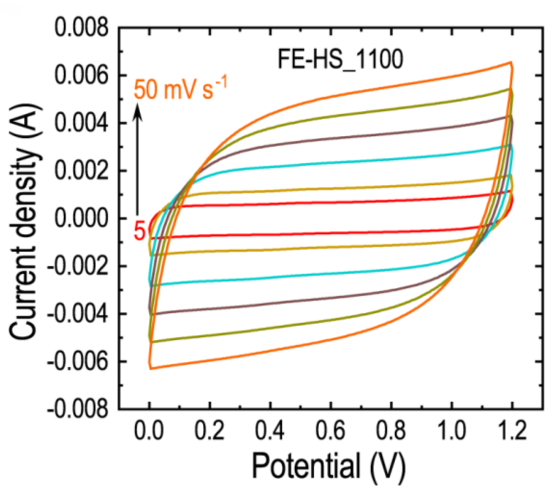

Figure S11: CV curves *vs*. scan rate of the symmetric supercapacitor prepared using FE-HS_1100 sample.

Table S1. Comparison of the electrochemical supercapacitance performance of the porous hollow carbon spheres with the other porous carbon materials including graphene and CNTs-based supercapacitors.

Sample	Electrolyte	Test System	Current density /Scan rate	Specific Capacitance (F g^{-1})	Ref.
Mesoporous carbon cubes derived from C_{70} cubes	1 M H_2SO_4	Three-electrode	1 A g^{-1}	205	[10]
Wheat straw-derived carbon	2 M NaOH	Three-electrode	2 mA cm^{-2}	162	[28]
Mango Seed husk carbon	2 M NaOH	Three-electrode	5 mA cm^{-2}	135	[30]
Chitin derived nitrogen-doped porous carbon	1 M H_2SO_4	Three-electrode	0.5 A g^{-1}	245	[31]
Sandwich-like chitosan porous carbon Spheres/MXene composite	1 M H_2SO_4	Three-electrode	0.5 A g^{-1}	362	[32]
Mesoporous carbon tubes derived from C_{60} tubes	1 M H_2SO_4	Three-electrode	5 mV s^{-1}	145.5	[33]
Mesoporous carbon rods derived from C_{60} rods	1 M H_2SO_4	Three-electrode	5 mV s^{-1}	132.3	[33]
Nanoporous carbon from "Konpeito-like C_{60} crystals"	1 M H_2SO_4	Three-electrode	5 mV s^{-1}	175	[34]
2D mesoporous carbon microbelts	1 M H_2SO_4	Three-electrode	1 A g^{-1}	290	[35]
Activated C_{70}	1 M H_2SO_4	Three-electrode	0.1 A g^{-1}	362	[36]
Mesoporous carbon tubes derived from C_{70} tubes	1 M H_2SO_4	Three-electrode	0.1 A g^{-1}	184.6	[37]
Wood-derived thick carbon	6 M KOH	Three-electrode	2 mA cm^{-2}	330.2	[62]
Porous graphene spheres	6 M KOH	Two-electrode	0.2 A g^{-1}	179	[63]
Graphene fiber	1 M H_2SO_4	Three-electrode	0.2 A g^{-1}	279	[64]
Graphene	$EMIMBF_4$	Two-electrode	1 A g^{-1}	154.1	[65]
Graphene	1 M H_2SO_4	Three-electrode	1 mV s^{-1}	226	[66]
RG-O /NH_2RG-O	1 M H_2SO_4	Three-electrode	2 mV s^{-1}	68	[67]
Graphene/Graphene oxide	6 M KOH	Two-electrode	0.5 A g^{-1}	189	[68]
N-doped Graphene	6 M KOH	Two-electrode	1 A g-1	405	[69]
N-doped Carbon	20 M LiTFSI	Two-electrode	0.1 A g^{-1}	167	[70]
N-doped Carbon	6 M KOH	Three-electrode	1 A g^{-1}	293 [1]	[71]
N-doped Carbon	0.5 M H_2SO_4	Three-electrode	1 A g^{-1}	855	[72]
Graphene/CNT fiber	PVA-KOH gel	Two-electrode	0.8 A g^{-1}	139	[73]
r-GO/CNT fiber	PVA-H_3PO_4 gel	Two-electrode	10 mV s^{-1}	39.3 F cm^{-3}	[74]
Activated C_{60}	1 M BMIM BF_4/AN	Two-electrode	0.2 A g^{-1}	118	[75]
N-doped activated C_{60}	1 M BMIM PF_6/AN	Two-electrode	1 A g^{-1}	114.6	
Mesoporous carbon tubes derived from macaroni C_{60} crystals	1 M H_2SO_4	Three-electrode	1 A g^{-1}	422	[76]
Hiearchically porous hollow carbon spheres derive from C_{60}-EDA hollow spheres	1 M H_2SO_4	Three-electrode	1 A g^{-1}	293	This work
	1 M H_2SO_4	Two-electrode	1 A g^{-1}	164	

Reference

63. Wei, J.; Luo, C.; Li, H.; Lv, W.; Liang, J.; Deng, Y.; Huang, Z.; Wang, C.; Kang, F.; Yang, Q.-H. Direct assembly of micron-size porous graphene spheres with a high density as supercapacitor materials. *Carbon* **2019**, *149*, 492-498.
64. Chen, S.; Ma, W.; Cheng, Y.; Weng, Z.; Sun, B.; Wang, L.; Chen, W.; Li, F.; Zhu, M.; Cheng, H.-M. Scalable non-liquid-crystal spinning of locally aligned graphene fibers for high-performance wearable supercapacitors. *Nano Energy* **2015**, *15*, 642-653.
65. Liu, C.; Yu, Z.; Neff, D.; Zhamu, A.; Jang, B. Z. Graphene-based supercapacitor with an ultrahigh energy density. *Nano Lett*. **2010**, *10*, 4863-4868.
66. Wu, Z.-S.; Sun, Y.; Tan, Y.-Z.; Yang, S.; Feng, X.; Müllen, K. Three-dimensional graphene-based macro- and mesoporous frameworks for high-performance electrochemical capacitive energy storage. *J. Am. Chem. Soc*. **2012**, *134*, 19532-19535.
67. Lai, L.; Yang, H.; Wang, L.; Teh, B. K.; Zhong, J.; Chou, H.; Chen, L.; Shen, Z.; Ruoff, R. S.; Lin, J. Preparation of supercapacitor electrodes through selection of graphene surface functionalities. *ACS Nano* **2012**, *6*, 5941-5951.
68. Xu, B.; Yue, S.; Sui, Z.; Zhang, X.; Hou, S.; Cao, G.; Yang, Y. What is the choice for supercapacitors: graphene or graphene oxide? *Energy Environ. Sci*. **2011**, *4*, 2826-2830.
69. Elessawy, N. A.; Nady, J. E.; Wazeer, W.; Kashyout, A. B. Development of high-performance supercapacitor based on a novel controllable green synthesis for 3D nitrogen doped graphene. *Sci. Rep*. 2019, 9, 1129.
70. Liu, X.; Mi, R.; Yuan, L.; Yang, F.; Fu, Z.; Wang, C.; Tang, Y. Nitrogen-doped multi-scale porous carbon for high voltage aqueous supercapacitors. *Front. Chem*. **2018**, 6, 475.

71. Sun, L.; Tian, C.; Fu, Y.; Yang, Y.; Yin, J.; Wang, L.; Fu, H. Nitrogen-doped porous graphitic carbon as an excellent electrode material for advanced supercapacitors. *Chem. Eur. J.* **2014**, *20*, 564-574.
72. Lin, T.; Chen, I-W.; Liu, F.; Yang, C.; Bi, H.; Xu, F.; Huang, F. Nitrogen-doped mesoporous carbon of extraordinary capacitance for electrochemical energy storage. *Science* **2015**, *350*, 1508-1513.
73. Park, H.; Ambade, R.B.; Noh, S.H.; Eom, W.; Koh, K.H.; Ambade, S.B.; Lee, W.J.; Kim, S.H.; Han, T.H. Porous graphene-carbon nanotube scaffolds for fiber supercapacitors. *ACS Appl. Mater. Interfaces* **2019**, *11*, 9011-9022.
74. Ma, W.; Li, M.; Zhou, X.; Li, J.; Dong, Y.; Zhu, M. Three-dimensional porous carbon nanotubes/reduced graphene oxide fiber from rapid phase separation for a high-rate all-solid-state supercapacitor. *ACS Appl. Mater. Interfaces* **2019**, *11*, 9283-9290.
75. Tan, Z.; Ni, K.; Chen, G.; Zeng, W.; Tao, Z.; Ikram, M.; Zhang, Q.; Wang, H.; Sun, L.; Zhu, X.; Wu, X.; Ji, H.; Ruoff, R.S.; Zhu, Y. Incorporating pyrrolic and pyridinic nitrogen into a porous carbon made from C60 molecules to obtain superior energy storage. *Adv. Mater*. **2017**, *29*, 1603414.
76. Maji, S.; Shrestha, R.G.; Lee, J.; Han, S.A.; Hill, J.P.; Kim, J.H.; Ariga, K.; Shrestha, L.K. Macaroni Fullerene Crystals-Derived Mesoporous Carbon Tubes as a High Rate Performance Supercapacitor Electrode Material. *Bull. Chem. Soc. Jpn.* **2021**, *94*, 1502-1509.
